# Supplementary material for: High-Performance Self-Powered Ultraviolet Photodetector Based on Nano-Porous GaN and CoPc p–n Vertical Heterojunction
Source: Nanomaterials (Basel). 2019 Aug 26;9(9):1198. doi: 10.3390/nano9091198 (PMC6780170; doi:10.3390/nano9091198)
Supplement: Supplementary file 1 [file nanomaterials-09-01198-s001.pdf]

Supporting Information:

Article

High-Performance Self-Powered Ultraviolet Photodetector Based on Nano-Porous GaN and CoPc *p–n* Vertical Heterojunction

Yan Xiao [1,‡], Lin Liu [2,3,‡], Zhi-hao Ma [1], Bo Meng [1], Su-Jie Qin [3,*], Ge-Bo Pan [2,*]

[1] Key Laboratory of Optoelectronic Devices and Systems of Ministry of Education and Guangdong Province, College of Optoelectronic Engineering, Shenzhen University, Shenzhen 518060, China
[2] Suzhou Institute of Nano-tech and Nano-bionics, Chinese Academy of Sciences, Suzhou 215123, China
[3] Department of Health and Environmental Sciences, Xi'an Jiaotong-Liverpool University, Suzhou 215123, China
* Correspondence: gbpan2008@sinano.ac.cn (G.-B.P.); sujie.qin@xjtlu.edu.cn (S.-J.Q.); Tel.: +86-0512-62872663 (G.-B.P. & S.-J.Q.)
‡ These authors contributed equally to this work.

Received: 31 July 2019; Accepted: 21 August 2019; Published: date

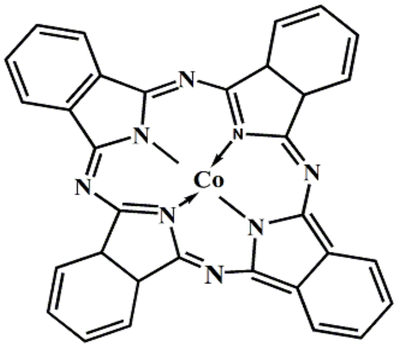

Figure S1. The chemical structure of CoPc.

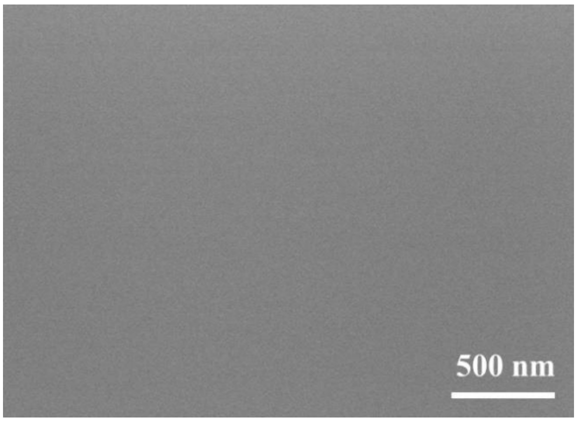

Figure S2. SEM images of pure flat-GaN.

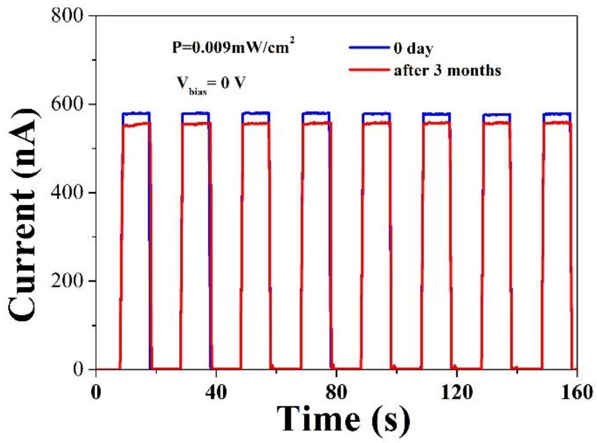

Figure S3. Time-dependent on/off switching of the device at day one and after 3 months under 365 nm light illumination, the light intensity is 0.009 mW/cm^2 and bias potential is 0 V.

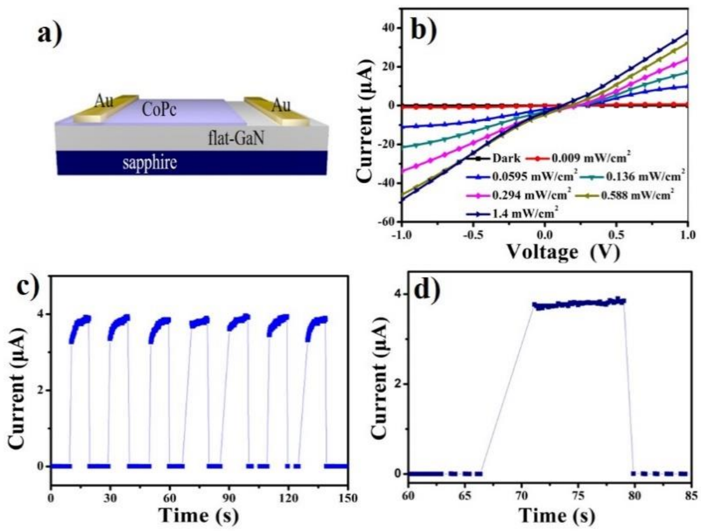

Figure S4. (a) Schematic of a PD device based on CoPc/flat-GaN p-n vertical heterojunction. (b) I-V curves of the PD under 365 nm light with different power density illumination. (c) Time-dependent on/off switching of the device under 365 nm light illumination, the light intensity is 1.4 mWcm^{-2} and bias potential is 0 V. (d) The enlarged portion of one response and the recover process.
